# What has the MEPI Program Taught Us?

**DOI:** 10.29024/aogh.3

**Published:** 2018-04-30

**Authors:** Roger I. Glass, Flora Katz, Ann Puderbaugh, Peter H. Kilmarx

**Affiliations:** 1 Fogarty International Center National Institutes of Health, US

The Medical Education Partnership Initiative, MEPI, has been an audacious program launched by The President’s Emergency Fund for AIDS Relief (PEPFAR), NIH and HRSA to improve medical education in a dozen African countries effected by the HIV/AIDS pandemic. It was established because the tremendous deficit in Africa of health-care professionals – from physicians and nurses to the entire group of allied health-care workers. For NIH, it also has evolved a new approach to the way we have traditionally supported research training in low- and middle-income countries. PEPFAR was born when President George W. Bush became convinced that the HIV/AIDS pandemic was a threat to the stability of Africa and the security of the United States. The emergency phase of the program set out to determine whether massive U.S. funding for HIV treatment and control could slow the pandemic, allowing longer-term solutions that would turn over greater leadership to African governments. Eric Goosby, the US Ambassador for PEPFAR, and Francis Collins, the NIH Director, reasoned that if the African governments were to take ownership of the epidemic tomorrow, they needed to build their own workforce and systems to deliver preventive and curative services for their populations today. MEPI focused on ramping up the training of physicians, teachers and researchers and developing health systems to deliver treatment long term, essentially care for life. Whenever foreign “emergency” health care workers could be replaced with local physicians and nurses, the cost of these programs could be reduced and trainees could assume greater leadership for the HIV health needs of the country. This initiative would also create jobs, save lives, improve health and worker productivity, and turn around the economies of the countries affected. Awards were offered exclusively to African institutions that had obtained government engagement and that had competed successfully through an NIH peer review process. The awardees could choose their own American collaborators as partners, a practice that reversed a long tradition of giving grants to U.S. institutions, which would then subcontract to their foreign partners. The African awardees had to demonstrate a track record of responsible grants management, a tradition of excellence in research, and a history of interacting with U.S. collaborators. This supplement of the *Annals of Global Health* documents some of the most interesting advances from the MEPI program. Several of these innovations deserve to be highlighted.

The concept of putting trust in the African investigators and their institutions to handle the $130 million-dollar program over a five-year period required careful monitoring and the ability to make in-course corrections. This task was handled by a separate grant made by HRSA to a partnership of a U.S. and an African Coordinating Center (CC). Over the period of the grant, the activities of the CC would shift from the U.S. to the African grantee, a transition that could lead to the longer-term sustainability of this effort with greater local leadership and continual improvement over time.

The first meeting of the MEPI program surprised us all in a most engaging way. Thirteen major grants were awarded for capacity building and 12 awards from NIH were linked to research training at the same institutions, so we expected 13 deans to be present. However, a number of grantees had reached out to include other medical schools in their proposals, so we were surprised and pleased that deans and representatives from about 40 African medical schools arrived, about one-fourth of all the medical schools in sub-Saharan Africa! Many of the African grantees concluded that if the goal of the program was to increase the quantity and quality of physicians being trained in-country, they should share the grants and engage other medical schools as partners of their program. Only a few of the principal investigators (PIs) knew each other before the program began but comradery in this shared mission grew rapidly.

The PIs have held one large annual meeting and a small PI council meeting each year to monitor progress of the program. As part of the annual evaluation, site visits were made to each school and PIs were included in the teams.By the end of the five-year program, the PIs had bonded and developed a common vision with the aspiration that the MEPI network would transform medical education in Africa and should continue regardless of whether the original grant was renewed.

A PI council set up working groups to address common themes. One group engaged in strengthening the medical curriculum, introducing simulation labs to give medical students experience with manikin models before engaging with patients, training at district hospitals so students could have more direct hands-on experience with patients, and working in inter-professional teams with nurses, pharmacists and other allied health students. Many medical students participated in research projects that addressed questions that could improve the delivery of care. Medical school libraries were transformed from dusty collections of old texts and poorly archived journals to 21st Century e-Libraries connected by broadband to Hanari, NLM and other websites, allowing immediate access to the most current medical literature. An eLearning working group spearheaded the introduction of tablet PCs to all medical students in the prime grantees institution, networked through hotspots on campus.

All schools established Research Support Centers to provide help with ethical review, grants administration, grant writing and information about research calls. During the five years of the program, more than 100 other grants from more than 11 NIH Institutes and Centers were awarded to the MEPI institutions, proof that the local investigators and their partners were able to compete for research grants on topics best studied in an African setting.

At Fogarty, we encouraged local governments to support the MEPI initiative but we not sure how this might happen. In Zimbabwe, the Minister of Education realized that the work of MEPI fell into his portfolio so he provided an additional $400,000 to his National Medical School to support the program. In Zambia, the MEPI PI convinced the Minister of Health to open a new state-funded medical school to accelerate the training of physicians with teachers who graduated from the MEPI program. In Tanzania, the Minister of Telecommunications visited the MEPI site at Moi University in Arusha and offered free high-speed broadband access for 10 years to facilitate the spread of their innovative eLearning curriculum around the country.

Most important, the PIs became so engaged with each other that they established AFREHealth, the African Forum for Research and Education in Health, as a way to sustain the advances made by the MEPI grantees and spread the positive outcomes throughout Africa schools of medicine, nursing and allied health sciences.

The MEPI program has been transformative for medical education in each country at the main institutions and in networks with their partners. However, the task of strengthening medical education, increasing the supply of physicians, nurses and allied health professionals does not occur overnight. MEPI has started the process which will require further investments over the next decade to continue building on the outstanding frameworks that have been established. New initiatives will be needed to sustain this momentum in the future bringing in funds from the government, the private sector, and the broader philanthropic and donor community.

What might have happened if we had not supported MEPI? Clearly, there would be an even greater delay in building the workforce needed to address the HIV pandemic. However, another value of the MEPI program has been its ability to address problems of global health security. When the Ebola outbreak began in 2014, MEPI partners stood ready. In Uganda, they were quick to stop the spread of an outbreak of another fatal viral disease caused by the Marburg virus on their own, and with no fanfare. In Ghana, grantees from a linked MEPI program training emergency health professionals screened people at border crossings from the Ebola-affected areas, providing a first line of defense against the introduction of the disease in country. In Nigeria, an NIH-funded grantee helped stop the immediate spread of Ebola from the first Ebola victim who arrived in Lagos, an intervention that prevented a major outbreak in the most populous country of Africa. And in those countries without a MEPI-style program – Sierra Leone, Guinea and Liberia – without robust lab facilities and a cadre of trained personnel, the Ebola outbreak spread wildly, requiring a multi-billion-dollar emergency response. Clearly, having skilled manpower on site with the ability to network with their peers had enormous benefits that could prevent outbreaks and save lives the next time a problem erupts. We need a MEPI-style program for these countries as well.

In summary, this supplement and the previous supplement in *Academic Medicine* document a few of the many lessons we have all learned from the MEPI experience. The supplement was initiated by the MEPI PIs to highlight their activities and the accomplishments of the program through their own eyes. The program to us has confirmed the huge and sustained value of these south-south and south-north networks, and the creative ability of African academic leaders to work together and build a program of their own which is much greater than the sum of its parts. This network, built over the past six years, needs to mature and continue into the future. It has sprouted a major African initiative to bring together African institutions of health, medicine, nursing and allied health sciences. AFREhealth can be a commons to share ideas, a clearing house of opportunities, an organization to provide leadership and best practices for teaching and research, and a place to innovate education of health professionals throughout sub-Saharan Africa. Many of these activities we could not have envisioned at the start of the program. Today, we are proud to see how well this experiment has worked. Giving these African leaders, these PIs, their own voice to work together, share experiences, set their own priorities and choose their own partners has been a formula that has succeeded. It will take years, if not decades, to fully develop this capacity. MEPI is a flagship that we hope will gather increased visibility from governments, the private sector and international funders to help sustain a program that has already contributed so much. The future of improved health in Africa and the world’s success in ending the pandemic of HIV/AIDS will depend upon it.

